# Assessment of the Anti-Cancer Efficiency of Silver *Moringa oleifera* Leaves Nano-extract against Colon Cancer Induced Chemically in Rats

**DOI:** 10.31557/APJCP.2021.22.10.3267

**Published:** 2021-10

**Authors:** Wael Mahmoud Aboulthana, Wafaa Ghoneim Shousha, Ehab Abdel-Raouf Essawy, Mahmoud Hosny Saleh, Alaa Hamed Salama

**Affiliations:** 1 *Biochemistry Department, National Research Centre, 33 El Bohouth St. (Former El Tahrir St.), Dokki, Giza, P.O. 12622, Egypt. *; 2 *Chemistry Department, Faculty of Science, Helwan University, Ain Helwan, Cairo, Egypt.*; 3 *Department of Pharmaceutics, Faculty of Pharmacy, Ahram Canadian University, 6* ^*th*^ * of October City, Cairo, Egypt. *; 4 *Pharmaceutical Technology Department, National Research Centre, 33 El Bohouth St. (Former El Tahrir St.), Dokki, Giza, P.O. 12622, Egypt. *

**Keywords:** Colorectal cancer, Moringa oleifera, green nanotechnology, isoenzymes, gene expression

## Abstract

**Background:**

Colorectal cancer (CRC) categorized as the most common type of gastrointestinal cancers affected both genders equally. Chemotherapeutic drugs became limited due to their deleterious side effects. Therefore, efficiency of *M. oleifera* leaves extract increased by incorporating silver nanoparticles (Ag-NPs) then studied against colon cancer induced by azoxymethane (AOM) in rats.

**Methods:**

Different hematological and biochemical measurements in addition to specific tumor and inflammatory markers were quantified. Histopathological examination for Colonic tissues was performed. Native proteins and isoenzyme patterns were electrophoretically detected in addition to assaying expression of Tumor Protein P53 (*TP53*) and Adenomatous Polyposis Coli (*APC*) genes in colonic tissues.

**Results:**

*M. oleifera *nano-extract restored levels of the hematological and biochemical measurements in addition to levels of tumor and inflammatory markers to normalcy in both of nano-extract simult- and post-treated groups. Also, it minimized severity of the histopathological alterations in the simult-treated group and prevented it completely in the post-treated group. The lowest similarity index (SI%) values were noticed with electrophoretic protein (SI=61.54%), lipid (SI=0.00%) and calcium (SI=75.00%) moieties of protein patterns, catalase (SI=85.71%), peroxidase (SI=85.71%), α-esterase (SI=50.00%) and β-esterase (SI=50.00%) isoenzymes in addition to altering the relative quantities of total protein and isoenzyme bands in colon of cancer induced group. Moreover, levels of *TP53* and *APC* gene expression increased significantly (P≤0.05) in colon cancer induced group. The nano-extract prevented the qualitative and quantitative alterations in the different electrophoretic patterns in addition to restoring levels of the gene expressions to normalcy in both of simult- and post-treated groups.

**Conclusion:**

*M. oleifera* nano-extract exhibited ameliorative effect against the biochemical, physiological and molecular alterations induced by AOM in nano-extract simult- and post-treated groups.

## Introduction

Cancer categorized as one of the leading causes of death worldwide. Behaviors of the lifestyle including smoking, poor diet, physical inactivity and reproductive changes increase susceptibility of the body to cancer incidence in less economically developed countries (Moorthi et al., 2011). It is largely occurred as a result of one or more of the genetic mutations that might be inherited or acquired (Armstrong et al., 2001). Shepherd and Sridhar (2003) postulated that cancer development is a complex multistage process, involving not only genetic change, but also due to hormonal changes, cocarcinogen and tumor promoter effects such as activation of proto oncogenes to oncogenes and inactivation of tumor suppressor genes.

Based on Globocan estimates, it was reported that colorectal cancer (CRC) is the third most lethal cancer affecting both genders equally in developed countries (Elamin et al., 2015). During the CRC pathogenesis, sequential and multistep progression of epithelial cells initiated to a cancerous state with defined precancerous intermediaries (Raju, 2008). At different stages of carcinogenesis, the specific oncogenes and tumor suppressor genes altered and this consequently leads to accelerating the cancer incidence (Luceri et al., 2000). There are two or three substances suitable to induce colon tumors. CRC induced chemically as a result of azoxymethan (AOM) injection within 7 - 9 months (Reddy and Maeura, 1984). The induction process accelerated after administrating an inflammatory agent in drinking water to murine models following AOM injection. Dextran sodium sulfate (DSS) is the most effective inflammatory agent suitable to accelerate CRC induction within a short period (Tanaka et al., 2003). Carbohydrate antigen (CA) 19-9 and carcinoembryonic antigen (CEA) used as indicators for a biological state of colon tissues and their levels are related to CRC progression (Tanaka et al., 2010). Therefore, they can be used for early CRC diagnosis. The tumor suppressor genes responsible for expression of Tumor Protein P53 and Adenomatous Polyposis Coli (APC) are susceptible to the genetic mutations that play an important role in CRC progression (Masri et al., 2002; Bachman et al., 2004).

Chemotherapy, radiotherapy, surgery, hormonal and targeted therapy belong to the modalities for cancer treatment. Most of these available therapeutic options have different side effects (Nounou et al., 2015 ; Kooti et al., 2017). Cancer treatment by mean of chemotherapy became limited due to the deleterious side effects and multidrug resistance (Moorthi et al., 2011). As reported earlier by Craig and Beck (1999), about 74% of known anti-cancer agents that currently used nowadays were derived from various plant species. Moringa oleifera (*M. oleifera*) Lam. (local name Sajna) belongs to the Moringaceae family (Jahn, 1988). *M. oleifera* leaves are considered as a potential source of active phyto-constituents such as total phenolics, vitamins (A, C and E), ascorbic acid oxidase, polyphenol oxidase and catalase in addition to the minerals (Khatun et al., 2003). It was found that these constituents exhibit antioxidant and scavenging activities against oxidative stress induced by reactive oxygen species (ROS) (Sudha et al., 2010 ; Sreelatha and Padma, 2011). *M. oleifera* leaves are rich in high protein content that is considered as an ideal source of essential amino acids such as methionine, cysteine, tryptophan and lysine. They exhibited good availability for intestinal absorption and rumen degradability of nitrogen as compared to soybean meal (Soliva et al., 2005).

Many reports have described the potential therapeutic values of *M. oleifera* extracts. They exhibited their anti-cancer, anti-diabetic, anti-rheumatoid arthritis, anti-fungal, anti-microbial activities (Chuang et al., 2007), anti-atherosclerotic (Chumark et al., 2008), anti-fertility, anti-depressant (Sathya et al., 2010) and diuretic effects (Biswas et al., 2012). Moreover, administration of *M. oleifera* extracts alone lead to significant increase in RBCs and Hb as compared to control group. Therefore, *M. oleifera* extract exhibited beneficial effect against mitigating anemia induced chemically in rats (Osman et al., 2012). Furthermore, the plant extracts attenuated brain dysfunction and brain damage (Kirisattayakul et al., 2013). It ameliorated the brain damage and toxicity of the cerebral cortex induced by lead injection (Owolabi et al., 2014). The studies focused recently on evaluation of *M. oleifera* extract efficacy with respect to tumor-suppressive activity, but not on the molecular basis of the tumor-suppressive activity (Budda et al., 2011 ; Leone et al., 2015).

Synthesis of metal nanoparticles (M-NPs) attracted the attention due to their different characteristics as compared to macrostructures (Priyadarshini et al., 2013). Synthesis of silver nanoparticles (Ag-NPs) by reduction of aqueous silver nitrate into Ag-NPs during the exposure to plant extracts can be easily monitored by using UV-visible spectrophotometer. The plant extracts with Ag-NPs exhibited good antioxidant activity at lower concentrations (Johnsona et al., 2014). Recently, it was found that incorporation of Ag-NPs in the plant extract increased the total phenolic compounds and total flavonoids. Therefore, silver plant nano-extracts showed a higher antioxidant and antimicrobial activity compared to plant extract alone or silver nitrate (Abdel-Aziz et al., 2014; Shousha et al., 2019 ; Aboulthana et al., 2019).

Most of the biologically active components absorbed slowly due to their high molecular weights which decreases their ability to cross the cellular membrane and hence decreases their efficacy and bioavailability. Therefore, the traditional medicinal plants integrated with nanotechnology to solve the stability problems for increasing their bioavailability and reducing their toxicity (Bonifácio et al., 2014; Mamillapalli et al., 2016). Development of metal plant nano-extracts showed great promise to solve their inherent stability problem (Rozenberg and Tenne, 2008). Incorporation of M-NPs into plant extracts increased the biological activities at lower concentrations as compared to plant extract solely and hence it increases the practical applications (Johnsona et al., 2014 ; Abdel-Aziz et al., 2014). From this point of view, the study was designed to develop novel strategy for CRC treatment by mean of green nanotechnology through incorporating Ag-NPs into *M. oleifera* leaves extract to enhance its antioxidant efficiency as suggested by Shousha et al., (2019) and supported by Aboulthana et al., (2021) then to evaluate nano-extract against CRC induced by AOM in rats. 

## Materials and Methods

*Silver M. oleifera Leaves Nano-extract Administration*

Based on our study published recently (Shousha et al., 2019), the aqueous silver *M. oleifera* nano-extract was prepared and characterized by advanced confirmatory technique and administrated orally by stomach tube at a dose of 687.50 mg/Kg body weight (b.wt) (1/20 of LD50).

*Induction of Colon Cancer in Rats*

Rats were injected by a single injection of AOM at a dose of 20 mg/kg b.wt interperitoneally (i.p.) to induce colon cancer then rats were given 2% DSS in drinking water for 7 days after one week of AOM injection (Yoshimi et al., 2009). Induction of the disease was detected starting from 8th week of AOM injection by histological examination. It was noticed that the disease induced after 12 weeks of AOM injection. Consequently, the induced rats were used to evaluate efficacy of the prepared silver *M. oleifera* nano-extract against induced colon cancer. 

*Experimental Design*

Healthy thirty-six adult male Wistar rats (weighting 120 - 150 g) were divided into six groups and housed in 6 cages (six per cage). Rats were provided with water ad libitum and maintained under normal nutritional and environmental conditions at 25 ± 2 °C. The experimental procedures were carried out based on the ethical protocol and guidelines approved by the institutional animal care of National Research Centre, Dokki, Giza, Egypt. 

Rats were randomly divided into Control group: Rats were fed with normal diet as ad libitum and received distilled water for 21 days, Silver *M. oleifera* nano-extract treated group: Rats were fed with normal diet associated with the treatment with *M. oleifera* nano-extract orally at a dose of 687.50 mg/Kg b.w. for 21 days, Colon cancer induced group: Rats were injected with AOM at a dose of 20 mg/kg i.p., Silver *M. oleifera* nano-extract pre-treated group: Rats were treated with *M. oleifera* nano-extract orally at a dose of 687.50 mg/Kg b.w. for 21 days followed by injecting AOM i.p. for another 12 weeks, Silver *M. oleifera* nano-extract simult.-treated group: Rats treated simultaneously with AOM i.p. and treated orally by *M. oleifera* nano-extract and Silver *M. oleifera* nano-extract post-treated group: Rats were injected AOM i.p. for 12 weeks followed by the treatment with *M. oleifera* nano-extract orally at a dose of 687.50 mg/Kg b.w. for another 21 days.

*Collection of Blood Samples and Tissues*

At end of the experimental period, rats were anaesthetized then the heparinized blood samples were collected from the retro orbital plexus for the hematological measurements. For biochemical measurements, the sera were collected by centrifuging blood samples at 4,000 rpm for 15 min. After sacrificing by decapitation, colon tissues were excised and washed in ice-cold saline. Each colon tissue was splitted into 2 portions. Portion (I) was immediately fixed in formal saline (10%) for histopathological examination. Portion (II) was immediately washed in physiological saline then homogenized in Tris-HCl buffer (0.01 M and pH 7.4). Aliquots of the clear supernatants were used for quantifying markers of the inflammatory response and oxidative stress and for assaying the native electrophoretic patterns.

*Hematological and Biochemical Measurements*

The hematological measurements including parameters of the complete blood count were quantified in heparinized blood samples using an automatic blood analyzer (ABX Micros 60 manufactured by HORIBA ABX SAS). The biochemical parameters (liver enzymes (alanine transaminase (ALT) and aspartate transaminase (AST), lipid (total cholesterol (TC) and triglycerides (T.Gs)), renal functions (urea and creatinin) and protein measurements (total protein and albumin)) were estimated in serum samples by colorimetric methods using the commercially available kits purchased from Spectrum Diagnostics Egyptian Company for Biotechnology (Cairo, Egypt).

*Tumor Markers*

The specific tumor markers (Carcinoembryonic Antigen (CEA) and Carbohydrate Antigen (CA) 19-9) that were released in case of colon cancer incidence were quantitatively assayed in sera of all treated groups using Enzyme Linked Immune Sorbent Assay (ELISA) technique.

*Inflammatory Markers*

The inflammatory markers that were expressed in response to incidence of colon cancer include C reactive protein (CRP) level and Myeloperoxidase (MPO) activity. The CRP level was determined in sera specimens and expressed as mg/L using commercially available enzymatic kit (Bio-Diagnostic, Cairo, Egypt). The MPO activity was quantified in supernatant of colon tissue homogenates and expressed by Δ/min/mg protein according to the method suggested by Correia et al., (2012).

*The Major Cellular Contents in Colon Tissues Homogenates*

In colon tissues homogenates, calcium concentration was determined using fully automated with microprocessor-controlled electrolyte system (Sensa Core’s ST-200 CL). Activity of lactate dehydrogenase (LDH) was measured according to the method described by Babson and Babson (1973). Glucose level was measured by enzymatic colorimetric method using commercial Kit (Bio-Diagnostic, Cairo, Egypt).

*Markers of Oxidative Stress*

These markers were assayed in clear supernatants of colon tissue homogenates. Lipid peroxidation product (LPO) was measured as malondialdehyde (MDA) which is the end product of peroxidation reaction and the result was expressed as nmol/g wet tissue (Ohkawa et al., 1979). Total protein carbonyl content (TPC) was quantified according to the method suggested by Levine et al. (1994) and the result was expressed as nmol of reactive carbonyl compounds per mg protein of tissue. Total antioxidant capacity (TAC), catalase (CAT) and glutathione peroxidase (GPx) activity were assayed according to the methods suggested by Koracevic et al. (2001) as mM/L, Aebi (1984) and Paglia and Valentine (1967) as Unit per gram of tissue, respectively.

*Statistical Analysis*

Data compiled in tables and illustrated figures were expressed as mean ± standard error (SE). They were analyzed statistically by one-way analysis of variance test (one-way ANOVA) using the Statistical Package for Social Sciences (SPSS for windows, version 11.0) followed by Bonferroni test as Post-Hoc. The difference between the groups at a “P” value of less than 0.05 was considered statistically significant.

*Histopathological Examination*

Specimens were autopsied from colonic tissues of different groups and immediately fixed in formal saline then studied histopathologically according to the protocol described by Banchroft et al., (1996). The washed tissue specimens were dehydrated in serial alcohol solutions and cleared in xylene then embedded in paraffin wax blocks. The tissue sections (at thickness 4 microns) were collected on glass slides and deparaffinized then stained by hematoxylin & eosin (H&E) stain to be examined under the light electric microscope. The histopathological changes were assigned and scored then rated between 0 (no damage) and +++ (maximal damage) (Dommels et al., 2007).

*Electrophoretic Assays*

Known weights of colon tissues (0.2 gm) were freezed rapidly in liquid nitrogen then homogenized in 1 ml water-soluble extraction buffer. The homogenates were centrifuged for 5 min at 10,000 rpm then the clear supernatants separated and transferred into another tubes. Equal volumes of the individual supernatants in each group were pooled together and used as one sample. Total protein concentration was measured in all pooled samples according to the method suggested by Bradford (1976) to ensure that concentration of the protein loaded in all wells equal during all electrophoretic assays. 

Native proteins were separated electrophoretically through Polyacrylamide Gel Electrophoresis (PAGE) then stained by commassie brilliant blue according to the method documented by Laemmli (1970) and improved recently by Darwesh et al., (2015). After electrophoretic run, native gels were stained for detecting lipid moiety of native protein by Sudan Black B (SBB) and the stained lipoprotein bands appeared as black bands (Subramaniam and Chaubal, 1990). For detecting calcium moiety of native protein, native gels were stained using alizarin Red “S”. The stained calcium moieties appeared as yellow bands (Zacharia and Kakati, 2004; Abd Elhalim et al., 2017; Abulyazid et al., 2017). For detecting the electrophoretic isoenzymes, electrophoretic catalase (CAT) and peroxidase (POX) patterns were assayed after electrophoretic run by incubating native gel with H2O2 as substrate then stained. The stained CAT subunits appeared as yellow bands (Siciliano and Shaw, 1976) and the stained POX subunits appeared as brown bands in the gel (Rescigno et al., 1997). The electrophoretic esterase (EST) pattern was carried out to localize in-gel EST activity by incubating native gel in conditioning buffer then staining in reaction mixture consisting of α, β-naphthyl acetate (5.58 X 10-3 mM, pH 7.5) as substrates along with dye coupler Fast Blue RR. The α-naphthyl acetate used as substrate for detecting α-esterase (α-EST) that appeared as brown bands and β-naphthyl acetate used as substrate for β-esterases (β-EST) that appeared as dark pink bands (Ahmad et al., 2012).

*Data Analysis*

The photographed PAGE plates were analyzed using Quantity One software (Version 4.6.2). This program used for determining the relative mobility (Rf), band percent (B%) and relative band quantity (Qty%) of the electrophoretically separated bands in addition to the molecular weights (Mwts) that were determined as compared to marker of standard molecular weights with regularly spaced bands ranging from 6.458 to 195.755 KDa. The similarity index (SI%) and genetic distance (GD%) were calculated according to the equation suggested by Nei and Li (1979).

*Molecular Assay*

*RNA Extraction and cDNA Synthesis*

Total RNA was extracted from colon tissues using easy-spinTM Total RNA Extraction Kit (iNTRON Biotechnology). Concentration and purity of the RNA yields were measured using NanoDrop^®^ (ND-1000 Spectrophotometer, NanoDrop Technologies Inc, Delaware, USA). Purity of the RNA yield was accepted in all samples (A260/A280 absorbance ratio of between 1.85 and 2.0). All RNA samples were normalized to 1,000 ng/μL, DNase treated (DNaseI, Thermoscientific) and reverse transcribed into First Strand cDNA using Power cDNA Synthesis Kit (iNTRON Biotechnology) according to manufacturers’ protocols.

*Primer Design*

Specific primers for APC (F: 5’-TGGAGAGAGAACGAGGTATT-3’ ; B: 5’-CTTCCATCACTTTGGCTATCT-3’) and tumor protein P53 (TP53) (F: 5’- ACTTTAGGGCTTGTTATGAGAG-3’ ; B: 5’-CAGGAACCAGTTTGCATAGA-3’) genes were designed on the bases of the Rattus norvegicus sequence Gene bank accessions NM_012499 and NM_030989 respectively using Primer Quest Tool and synthesized by Macrogen company. The designed primers were used for Real time quantitative PCR (qRT-PCR). For gene expression analysis, Beta actin (Actb) was used as endogenous control and its primers (F: 5’-TGTGGATTGGTGGCTCTATC-3’; B: 5’-CAGTAACAGTCCGCCTAGAA-3’) were designed based on sequence of Gene bank NM_031144.

*Gene Expression Using qRT-PCR*

Expression of the *APC *and *TP53* genes were determined in colon tissues of control and different treated groups. A master mix was prepared for each assay containing 10μL of 2X SYBR green PCR mix (TOPreal™ qPCR 2X PreMIX, enzynomics), 1 ul of 10uM primer sets and nuclease-free water. For each sample, a 25 μL reaction volume of mastermix containing 1 ul of cDNA was prepared. The qPCR was performed by Qiagen’s real-time PCR system (Rotor-Gene Q) using default cycling conditions : 40 cycles of an initial activation step of 95°C for 15 min followed by 95°C for 10 s (melt), 57°C for 15s (anneal) and 72°C for 30s (extend). The mean Cycle threshold (Ct) values were calculated and then normalized to Actb using ΔCt method. Changes in relative expression were calculated using the 2^-ΔΔCt^ method [K.J.Livak ,T.D. Schmittgen , methods 25(4)(2001)402-408].

*Statistical Analysis*

Molecular data were statistically analyzed using the SPSS computer program (SPSS Inc., Chicago, Illinois, USA). The non-parametric Mann-Whitney test used to compare levels of the relative gene expression in all treated groups to control. Results were reported as the arithmetic mean ± SE. The “P” value less than 0.05 is considered statistically significant (***=P<0.001, **=P<0.01, *=P<0.05 and ns=statistically non- significant).

## Results

Although the study was designed to reveal efficiency of *M. oleifera* leaves extract after incorporating Ag-NPs against AOM induced colon cancer, it was found that AOM caused severe alterations at the hematological and biochemical levels during induction of the disease.

*Hematological Measurements*

As depicted in Table 1, AOM caused no significant alterations in hematological measurements related to indices of red blood cells (red blood cells (RBCs), hemoglobin (HB), hematocrit (HCT), corpuscular volume (MCV), mean corpuscular haemoglobin (MCH), mean corpuscular haemoglobin concentration (MCHC). As regard to red blood cell distribution width (RDW), mean platelet volume (MPV), platelet count (PLT), it was found that AOM caused significant (P≤0.05) elevation in levels of these measurements. Nano-extract could not affect RDW level in pre-treated group when compared to AOM induced colon cancer group but it decreased RDW level significantly (P≤0.05) and restored its level to normal values in the simult- and post-treated groups. It decreased levels of MPV and PLT significantly (P≤0.05) in all nano-extract treated groups as compared to colon cancer induced group and restored their levels to normalcy in the simult- and post-treated groups. On the other hand, it was noticed that AOM caused significant (P≤0.05) elevation in white blood cells (WBCs) and its differential count (lymphocytes, monocytes and granulocytes) when compared to control group. Nano-extract decreased these measurements significantly (P≤0.05) and restored their levels to normal values in all nano-extract treated groups.

*Biochemical Measurements*

As reported in Table 2, it was found that activities of liver enzymes (ALT, AST and ALP) in addition to TC and T.Gs levels increased significantly (P≤0.05) in sera of colon cancer induced group as compared to control group. The nano-extract at all therapeutic modes decreased their levels significantly (P≤0.05) as compared to colon cancer induced group and restored them to normalcy. Moreover, AOM affected kidney functions through increasing levels of urea and creatinin and decreasing levels of T.P and albumin significantly (P≤0.05). The nano-extract exhibited the same therapeutic effect in all nano-extract treated groups.

*Tumor Markers*

**Figure 1:**
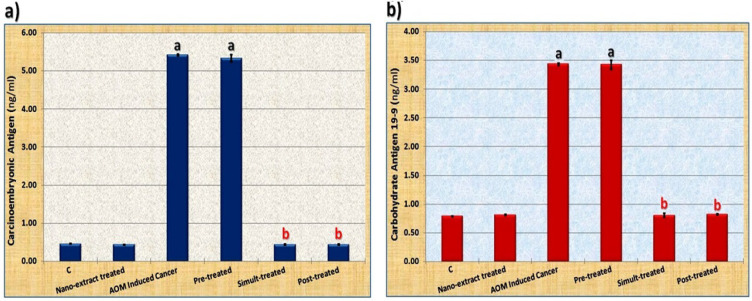
Data Showing the Ameliorative Effect *M. oleifera* Leaves Extract Extract after Incorporating Silver Nanoparticles (Ag-NPs) against Azoxymethane (AOM) Induced Colon cancer on a) Carcinoembryonic Antigen (CEA) and b) Carbohydrate Antigen (CA) 19-9 in sera of rats

The current study was concerned with assaying CEA and CA 19.9 that are considered as the most specific markers for colon cancer. It was found that levels of these tumor markers elevated significantly (P≤0.05) in AOM induced colon cancer group as compared to control group (Figure 1). The nano-extract declined their levels significantly (P≤0.05) in all nano-extract treated groups as compared to colon cancer induced group but it restored their levels to normal levels in the nano-extract simult- and post-treated groups.

*Inflammation Markers*

**Figure 2:**
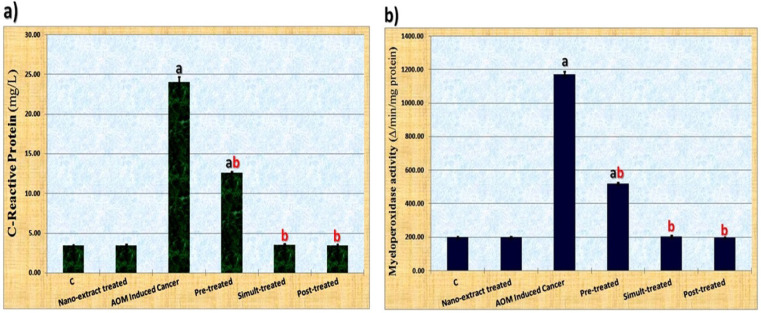
Data Showing the Ameliorative Effect of *M. oleifera* Leaves Extract after Incorporating Silver Nanoparticles (Ag-NPs) against Azoxymethane (AOM) Induced Colon Cancer on a) C Reactive Protein (CRP) in serum samples and b) Activity of Myeloperoxidase (MPO) in colon tissue homogenates of rats. Values expressed as mean ± SE, a: significant difference (P≤0.05) from control group, b: significant difference (P≤0.05) from toxic group

As regard to the inflammatory response, CRP is considered as the most common marker of the inflammatory reactions. Also, MPO activity categorized as the most specific inflammatory marker expressed as a result of colon cancer induction. Data illustrated in Figure 2 showed that AOM caused significant (P≤0.05) elevation in levels of these inflammatory markers (CRP level and MPO activity) as compared to control group. Although *M. oleifera* nano-extract caused significant (P≤0.05) decrease in levels of these inflammatory markers in all nano-extract treated groups as compared to colon cancer induced group, it restored levels of these measurements to normal values in nano-extract simult- and post-treated groups.

*Major Colonic Contents and Oxidative Stress Markers*

As regard to the assays that were carried out in supernatants of colon tissues homogenates, glucose and calcium in addition to activity of LDH enzyme are considered as the major cellular contents in colon tissues. Data recorded in Table 3 showed that glucose level and LDH activity declined significantly (P≤0.05) in colon cancer induced group associated with significant (P≤0.05) elevation in calcium level as compared to control group. As compared to colon cancer induced group, nano-extract caused significant (P≤0.05) increase in glucose and calcium levels in all nano-extract treated groups but it restored their levels to normalcy in nano-extract simult- and post-treated groups. As regard to, it decreased the LDH activity significantly (P≤0.05) and restored its activity to normal value in nano-extract simult- and post-treated groups.

Markers of the oxidative stress were represented by total antioxidant capacity (TAC) and activities of the antioxidant enzymes (CAT and GPx) in addition to products of the peroxidation reactions (LPO and TPC) that were assayed in colon tissues homogenates. Data recorded in Table 3 showed that AOM caused significant (P≤0.05) decrease in the TAC level and activities of the antioxidant enzymes associated with significant (P≤0.05) elevation in concentrations of the peroxidation products when compared to control group. Nano-extract didn’t show significantly changes in all markers of the oxidative stress in nano-extract pre-treated group when compared to colon cancer induced group. In the nano-extract simult- and post-treated groups, nano-extract caused significant (P≤0.05) increase in TAC level and activities of CAT and GPx associated with lowering concentrations of LPO and TPC as compared to colon cancer induced group and restored their levels to normalcy.

*Histopathological Examination*

**Figure 3:**
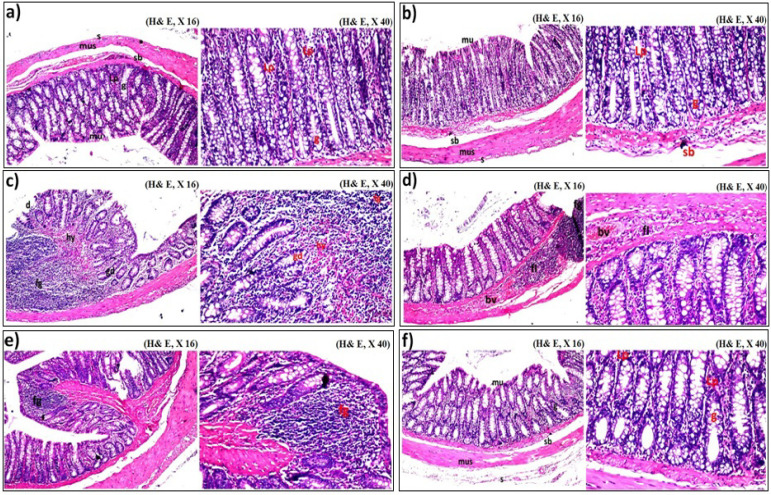
Histopathological Examination Showing the Ameliorative Effect of* M. oleifera* Leaves Extract after Incorporating Silver Nanoparticles (Ag-NPs) against Azoxymethane (AOM) Induced Colon Cancer on Colon Tissue of Rats. It revealed that a) control group, b) *M. oleifera* nano-extract treated group, c) AOM induced colon cancer group, d) *M. oleifera* nano-extract pre-treated group, e) *M. oleifera* nano-extract simult-treated group and f) *M. oleifera *nano-extract post-treated group

As revealed in colon of control rats (Figure 3a), it was found that there was no histopathological alteration and the normal histological structure of the mucosa layer with lining epithelium, lamina propria and glands as well as the underlying submucosa, muscularis and serosa were noticed. In *M. oleifera* nano-extract treated group (Figure 3b), there was no histopathological alteration and no deviation recorded from control group. As regard to colon cancer induced group, there were focal desquamation in the tips of the lining mucosal epithelium with massive inflammatory cells aggregation in the hyalinized lamina propria of the mucosa as in the submucosa in association with disfiguration of the glandular structure (glandular dysplasia) (Figure 3c). Colon cancer induced group showed the highest degree of inflammatory responses (+++ ; 75-100 %) and mild degree of disfiguration of the glandular histological structure (+ ; 25-50 %). In nano-extract pre-treated group, focal massive inflammatory cells aggregations as well as infilteration with congestion in the blood vessels were detected in the lamina propria of the mucosa and submucosa (Figure 3d). In nano-extract simult-treated group, the lamina propria of the mucosa as well as the submucosa showed focal inflammatory cells aggregations (Figure 3e). While in nano-extract post-treated group, there were no histopathological alterations as presented in Figure 3f. Therefore, the nano-extract minimized severity of the inflammatory responses that is considered as criteria of malignancy and stage of colon cancer in the nano-extract simult-treated group and prevented it completely in the post-treated group as compared to the other nano-extract treated ones.

*Electrophoretic Assays*

*Electrophoretic Protein Pattern*

As presented in Figure 5a, the native protein pattern was represented electrophoretically in colon of control rats by 7 bands identified at Rfs 0.16, 0.25, 0.59, 0.65, 0.76, 0.92 and 0.96 (Mwts 112.18, 75.66, 22.18, 19.45, 16.16, 7.51 and 6.04 KDa ; B% 9.97, 10.86, 18.56, 14.30, 14.56, 16.67 and 15.09 ; Qty% 9.80, 10.67, 17.19, 5.68, 19.26, 11.35 and 6.56, respectively). Four common bands were identified at Rfs 0.16, 0.25, 0.92 and 0.96. No characteristic bands were noticed. The nano-extract alone caused no alterations in native protein pattern and no deviation detected from the control group. AOM caused physiological alterations in the native protein pattern represented by hiding 3 normal protein bands with existence of 2 abnormal ones identified in AOM induced colon cancer group at Rfs 0.41 and 0.74 (Mwts 37.04 and 16.78 KDa ; B% 17.45 and 19.48 ; Qty% 15.98 and 15.34, respectively). 

The nano-extract showed no obvious changes from the AOM induced colon cancer group and the qualitative alterations still noticed in the nano-extract pre-treated group. Therefore, the lowest SI and highest GD values were recorded in AOM induced colon cancer and nano-extract pre-treated groups (SI=61.54% ; GD=38.46%). While in the other nano-extract treated groups, the nano-extract exhibited improvement in protein pattern by hiding the abnormal bands and restoring the normal ones (3 normal bands) that were identified in nano-extract simult-treated group at Rfs 0.57, 0.63 and 0.78 (Mwts 23.66, 19.97 and 15.86 KDa ; B% 16.66, 14.59 and 14.65 ; Qty% 18.79, 6.03 and 8.53, respectively) and identified in post-treated group at Rfs 0.56, 0.63 and 0.77 (Mwts 24.00, 19.82 and 15.98 KDa ; B% 19.34, 14.60 and 15.45 ; Qty% 14.83, 6.83 and 15.89, respectively). Therefore, the SI values increased and reached the highest value and hence the GD value decreased in that groups (SI=100.00% ; GD=0.00%).

From the SI% values, it was found that AOM induced colon cancer and nano-extract pre-treated groups were physiologically similar to control by 61.54%. Both of the nano-extract simult- and post-treated groups were qualitatively similar to control by 100%. AOM induced colon cancer group was similar to nano-extract pre-treated group by 100% and similar to the other nano-extract treated groups by 61.54%. As regard to the quantitative level, it was found that the relative quantities of the total protein bands declined significantly (P≤0.05) in colon cancer induced group as compared to control group (Table 4). The nano-extract increased the relative quantities significantly (P≤0.05) in all nano-extract treated groups as compared to colon cancer induced group and restored their values to normal level in nano-extract simult- and post-treated groups. 

*Electrophoretic Lipid Moiety of Native Protein Pattern*

As revealed in Figure 5b, the lipid moiety of native protein pattern was represented electrophoretically in colon of control rats by 2 bands identified at Rfs 0.07 and 0.74 (B% 47.95 and 52.05 and Qty% 14.93 and 24.52, respectively). No common bands were identified. Two characteristic (unique) bands were noticed. One of them was identified in the colon cancer induced group at Rf 0.15 (B% 100.00 and Qty% 19.64) and the other was identified in the nano-extract pre-treated group at 0.50 (B% 50.97 and Qty% 27.97). The nano-extract alone caused no alterations in the native lipoprotein pattern and no deviation detected from the control group. AOM caused physiological alterations in lipid moieties of native protein represented by hiding the normal bands with existence of characteristic one identified at Rf 0.15 (B% 100.00 and Qty% 19.64) in colon cancer induced group. Therefore, the lowest SI and highest GD values were recorded in colon cancer induced group (SI=0.00% ; GD=100.00%).

The nano-extract exhibited improvement in lipid moiety of native protein pattern by restoring one normal band identified at Rf 0.08 (B% 49.03 and Qty% 14.01) in nano-extract pre-treated group with existence of one abnormal band at Rf 0.50 (B% 50.97 and Qty% 27.97). So, the SI value increased with decreasing the GD value (SI=50% ; GD=50%) in that group. In the other nano-extract treated groups, the nano-extract exhibited physiological improvement through hiding the abnormal band with restoring the normal bands (2 bands) that were identified in nano-extract simult-treated group at Rfs 0.07 and 0.73 (B% 46.67 and 53.33; Qty% 13.53 and 18.03, respectively) and identified in the post-treated group at Rfs 0.05 and 0.73 (B% 46.72 and 53.28; Qty% 10.19 and 21.51, respectively). Therefore, the SI value increased and reached the highest value and hence the GD value decreased in that groups (SI=100.00% ; GD=0.00%).

From the SI% values, it was found that there was no similarity (SI=0.00%) between AOM induced colon cancer group and control group. Both of the nano-extract simult- and post-treated groups were qualitatively similar to control by 100%. While the nano-extract pre-treated group was similar to control by 50%. There was no similarity (SI=0.00%) when colon cancer induced group compared to all nano-extract treated, groups. At quantitative level, the relative quantities of the total lipoprotein bands declined significantly (P≤0.05) in colon cancer induced group as compared to control group (Table 4). The nano-extract increased the relative quantities significantly (P≤0.05) when compared to colon cancer induced group and restored quantities of the total bands to normal values in all nano-extract treated groups.

*Electrophoretic Calcium Moiety of Native Protein Pattern*

Data illustrated in Figure 5c showed that calcium moiety of native protein pattern was represented in control colon by 3 common bands identified at Rfs 0.12, 0.23 and 0.64 (B% 36.85, 31.47 and 31.67 and Qty% 9.81, 6.93 and 10.46, respectively). Only one characteristic (unique) band was identified in colon cancer induced group at Rf 0.34 (B% 20.06 and Qty% 8.33). The *M. oleifera* nano-extract alone caused no deviation detected in this protein pattern from the control group. AOM caused physiological alterations in calcium moieties of native protein pattern represented by appearance of 2 abnormal bands identified in colon cancer induced group at Rfs 0.34 and 0.89 (B% 20.06 and 18.04; Qty% 8.33 and 10.71, respectively). Therefore, the SI value decreased with increasing GD values in colon cancer induced group (SI=75.00% ; GD=25.00%).

The nano-extract could not prevent or minimize the qualitative alterations induced by AOM in nano-extract pre-treated group. On the contrary, the changes became severe and represented by hiding normal band with existence of abnormal one identified at Rf 0.90 (B% 30.04 ; Qty 13.16). Therefore, the lowest SI and highest GD values were recorded (SI=66.67% ; GD=33.33%) in that group. In the other nano-extract treated groups, the nano-extract exhibited physiological improvement through hiding the abnormal bands (2 abnormal bands) with restoring the normal one that identified in the nano-extract simult-treated group at Rf 0.65 (B% 31.11 ; Qty% 19.28) and identified in post-treated group at Rf 0.65 (B% 33.01 ; Qty% 16.49). Therefore, the SI values increased and reached the highest value and hence the GD values reached the lowest in that groups (SI=100.00% ; GD=0.00%).

From the SI% values, AOM induced colon cancer and nano-extract pre-treated groups were physiologically similar to control by 75.00 and 66.67%, respectively. Both of the nano-extract simult- and post-treated groups were similar to control by 100%. The colon cancer induced group was similar to nano-extract pre-, simult- and post-treated groups by 75.00%. At the quantitative level, it was noticed that the relative quantities of the total bands increased significantly (P≤0.05) in colon cancer induced group as compared to control group (Table 4). The nano-extract decreased the relative quantities significantly (P≤0.05) with respect to colon cancer induced group and restored quantities of the total bands to normal values in all nano-extract treated groups. 

*Electrophoretic Isoenzymes*

*Electrophoretic Catalase Pattern*

As presented in Figure 6a, it was noticed that CAT isoenzyme pattern was represented electrophoretically in colon of control rats by 4 types identified at Rfs 0.12, 0.56, 0.91 and 0.96 (B% 26.21, 26.27, 25.45 and 22.08 ; Qty% 17.24, 12.57, 6.39 and 3.43, respectively). Three CAT types (CAT2, CAT3 and CAT4) were considered as common bands. The *M. oleifera* nano-extract alone caused no abnormalities or deviation from control group. AOM caused physiological changes in the CAT pattern represented by hiding the CAT1 type without appearance of abnormal ones. Therefore, the SI decreased associated with increasing GD values in colon cancer induced group (SI=85.71% ; GD=14.29%).

The nano-extract could not prevent or minimize the qualitative alterations induced by AOM in the nano-extract pre-treated group. On the contrary, the changes became severe and represented by hiding the CAT1 type with existence of 2 abnormal (characteristic) bands identified at Rfs 0.34 and 0.67 (B% 19.87 and 18.08 ; Qty 6.85 and 5.19, respectively). Therefore, the lowest SI and highest GD values were recorded in that group (SI=66.67% ; GD=33.33%). In the other nano-extract treated groups, the nano-extract exhibited physiological improvement by restoring the CAT1 type (normal type) that identified in nano-extract simult-treated group at Rf 0.13 (B% 25.47 ; Qty% 10.84) and identified in post-treated group at Rf 0.12 (B% 25.44 ; Qty% 10.47). Therefore, the SI values increased and reached the highest value with decreasing the GD values in that groups (SI=100.00% ; GD=0.00%).

From the SI% values, it was found that the induced colon cancer and nano-extract pre-treated groups were qualitatively similar to control by 85.71 and 66.67%, respectively. Both of the nano-extract simult- and post-treated groups were similar to control by 100%. The colon cancer induced group was similar to the nano-extract pre-, simult- and post-treated groups by 75.00, 85.71 and 85.71%, respectively. As regard to the quantitative level, it was found that the relative quantities of the total CAT types decreased significantly (P≤0.05) in colon cancer induced group as compared to control group (Table 4). The nano-extract increased the relative quantities of the total CAT isoenzymes significantly (P≤0.05) when compared to colon cancer induced group and restored their values to normal levels in all nano-extract treated groups.

*Electrophoretic Peroxidase Pattern*

As revealed in Figure 6b, it was noticed that POX isoenzym pattern was represented in control colon by 4 types identified at Rfs 0.01, 0.30, 0.57 and 0.96 (B% 25.96, 22.44, 23.43 and 28.17 and Qty% 5.13, 7.14, 6.68 and 8.04, respectively). Two POX types (POX1 and POX4) are considered as common bands. The nano-extract alone caused no abnormalities or deviation from control group. AOM caused physiological changes in the POX pattern represented by hiding the POX3 type. Therefore, the SI decreased associated with increasing GD values in colon cancer induced group (SI=85.71% ; GD=14.29%). 

The nano-extract could not prevent or minimize the qualitative alterations induced by AOM in the nano-extract pre-treated group. On the contrary, it increased severity of the abnormalities that represented by hiding the POX3 type with existence of one abnormal (characteristic) band identified at Rf 0.41 (B% 21.99 ; Qty 7.56). Therefore, the lowest SI and highest GD values were recorded in that group (SI=75.00%; GD=25.00%). In the other nano-extract treated groups, the nano-extract exhibited physiological improvement by restoring the POX3 type (normal type) that identified in nano-extract simult-treated group at Rf 0.57 (B% 22.53 ; Qty% 7.37) and identified in the post-treated group at Rf 0.57 (B% 23.66 ; Qty% 4.41). Therefore, the SI values increased and reached the highest value and hence the GD values reached the lowest values in that groups (SI=100.00%; GD=0.00%).

From the SI% values, it was found that induced colon cancer and nano-extract pre-treated groups were qualitatively similar to control by 85.71 and 75.00%, respectively. Both of the nano-extract simult- and post-treated groups were similar to control by 100%. The induced colon cancer group was similar to nano-extract pre-, simult- and post-treated groups by 57.14, 85.71 and 85.71%, respectively. At the quantitative level, it was emphasized that the relative quantities of the total POX types decreased significantly (P≤0.05) in colon cancer induced group as compared to control group (Table 4). The nano-extract increased the relative quantities of the total POX isoenzymes significantly (P≤0.05) when compared to colon cancer induced group and restored their values to normal levels in all nano-extract treated groups.

*Electrophoretic Esterase Pattern*

As illustrated in Figure 6c, it was noticed that electrophoretic α-EST isoenzyme was represented in control colon by 2 types identified at Rfs 0.06 and 0.27 (B% 52.00 and 48.00 and Qty 15.34 and 20.96, respectively). The *M. oleifera* nano-extract alone caused no deviation from control group. No common bands were detected. Two characteristic (unique) bands were noticed. One of them was identified in colon cancer induced group at Rf 0.43 (B% 47.82 and Qty% 14.92) and the other identified in nano-extract pre-treated group at Rf 0.51 (B% 50.16 and Qty% 19.35). AOM caused qualitative abnormalities in the α-EST pattern represented by hiding the α-EST2 type. Therefore, the SI decreased associated with increasing GD values in colon cancer induced group (SI=50.00% ; GD=50.00%).

Nano-extract could not prevent or minimize the physiological alterations induced by AOM in the nano-extract pre-treated group. The changes were represented by hiding the α-EST1 type with existence of one abnormal (characteristic) band identified at Rf 0.51 (B% 50.16 and Qty% 19.35). Therefore, the SI% and GD% values were not changed as compared to colon cancer induced group (SI=50.00% ; GD=50.00%). In the other nano-extract treated groups, the nano-extract exhibited qualitative improvement by restoring the normal α-EST2 type that identified in the nano-extract simult-treated group at Rf 0.27 (B% 46.07 ; Qty% 27.79) and identified in the post-treated group at Rf 0.27 (B% 48.35 ; Qty% 17.53). This was associated with hiding the characteristic band. Therefore, the SI values increased and reached the highest value and hence the GD values reached the lowest values in that groups (SI=100.00% ; GD=0.00%).

From the SI% values, it was found that induced colon cancer and nano-extract pre-treated groups were qualitatively similar to control by 50.00%. Both of the nano-extract simult- and post-treated groups were similar to control by 100%. The induced colon cancer group was similar to the nano-extract simult- and post-treated groups by 50.00%. There was no similarity (SI=0.00%) between the colon cancer induced group and nano-extract pre-treated group. As regard to the quantitative level, it was found that AOM caused no significant changes in the relative quantities of the total α-EST types and values of the relative quantities of total α-EST types were approximately equal in all nano-extract treated groups as compared to control group and colon cancer induced group (Table 4).

As presented in Figure 6d, it was noticed that electrophoretic β-EST isoenzyme was represented in colon of control rats by 2 types identified at Rfs 0.17 and 0.42 (B% 44.24 and 55.76 and Qty% 25.79 and 40.62, respectively). The second β-EST type (β-EST2) is considered as common band. No characteristic bands were identified. The nano-extract alone caused no deviation from control group. In colon cancer induced group, various qualitative alterations were detected through hiding the β-EST1 type with appearance of one abnormal band identified at Rf 0.81 (B% 48.03 and Qty% 36.10). Therefore, the SI decreased associated with increasing GD values in that group (SI=50.00% ; GD=50.00%).

The nano-extract could not ameliorate the qualitative alterations induced by AOM in the nano-extract pre-treated group. The changes were represented by hiding the β-EST1 type with existence of one abnormal (characteristic) band identified at Rf 0.81 (B% 47.53 and Qty% 32.02). The SI% and GD% values were not changed as compared to colon cancer induced group (SI=50.00% ; GD=50.00%). In the other nano-extract treated groups, it improved the β-EST pattern by hiding the abnormal band with restoring the normal β-EST1 type that identified in the nano-extract simult-treated group at Rf 0.17 (B% 48.87; Qty% 25.99) and identified in the post-treated group at Rf 0.15 (B% 37.60 ; Qty% 24.98). Therefore, the SI values increased and reached the highest value with decreasing the GD values in that groups (SI=100.00% ; GD=0.00%). 

From the SI% values, it was found that the induced colon cancer and nano-extract pre-treated groups were qualitatively similar to control by 50.00%. Both of the nano-extract simult- and post-treated groups were similar to control by 100%. The colon cancer induced group was similar to the nano-extract pre-, simult- and post-treated groups by 100.00, 50.00 and 50.00%, respectively. There was complete similarity (SI=100.00%) between colon cancer induced group and nano-extract pre-treated group. At the quantitative level, it was fund that the relative quantities of the total β-EST types decreased significantly (P≤0.05) in colon cancer induced group as compared to control group. The nano-extract increased the relative quantities significantly (P≤0.05) in all nano-extract treated groups with respect to colon cancer induced group and restored quantities of the total β-EST types to normal values (Table 4). 

*Molecular assay*

As illustrated in Figure 6a, the colon cancer induced group showed highly significant increase (P=0.008) in level of *TP53* gene expression as compared to control group. In addition, levels of *TP53* gene expression were slightly significantly (P=0.026) higher in nano-extract pre-treated group as compared to control group. While in the other nano-extract treated groups, the simult- and post-treated groups didn’t show significant change in level of *TP53* gene expression than control group (P=0.519 and P=0.478, respectively).

As revealed in Figure 6b, the colon cancer induced group showed highly significant (P=0.010) increase in level of *APC* gene expression as compared to control group. In addition, levels of *APC* gene expression were significantly (P=0.037) higher in the nano-extract pre-treated group in comparison to control group. While the nano-extract simult- and post-treated groups didn’t show significant change in level of *APC* gene expression than control group (P=0.120 and P=0.107, respectively).

**Table 1:** Effect of M. oleifera Leaves Extract after Incorporating Silver Nanoparticles (Ag-NPs) against Azoxymethane (AOM) Induced Colon Cancer on Different Hematological Measurements in Rats

		C	*M. oleifera*	Induced cancer	*M. oleifera* nano-extract		
			nano-extract		Pre-treated	Simult-treated	Post-treated
Formed Elements	RBCs (106/ul)	5.86 ± 0.18	5.72 ± 0.27	5.78 ± 0.19	5.87 ± 0.17	5.76 ± 0.13	5.82 ± 0.12
	HB (g/ dl)	12.13 ± 0.36	12.45 ± 0.32	12.34 ± 0.24	12.76 ± 0.20	12.52 ± 0.14	12.53 ± 0.17
	HCT (%)	33.32 ± 1.08	34.92 ± 0.93	32.29 ± 1.16	33.04 ± 0.65	33.24 ± 0.60	34.10 ± 0.58
	MCV (um^3^)	56.90 ± 1.02	57.64 ± 1.20	54.62 ± 1.02	55.40 ± 1.08	56.57 ± 1.37	57.07 ± 1.18
	MCH (pg)	20.97 ± 0.38	21.51 ± 0.49	21.29 ± 0.55	21.29 ± 0.54	22.17 ± 0.49	21.84 ± 0.46
	MCHC (g/ dl)	36.46 ± 0.41	36.03 ± 0.67	34.04 ± 0.98	33.04 ± 0.73	33.96 ± 0.49	34.19 ± 0.64
	RDW (%)	15.62 ± 0.39	15.76 ± 0.19	20.24 ± 0.18^a^	20.02 ± 0.31^a^	15.40 ± 0.11^b^	15.18 ± 0.11^b^
	MPV (um^3^)	6.16 ± 0.15	6.02 ± 0.12	9.18 ± 0.10^a^	8.22 ± 0.11^ab^	6.13 ± 0.11^b^	6.05 ± 0.17^b^
	PLT (10^3^/ul)	446.30 ± 5.64	451.00 ± 7.11	849.10 ± 15.13^a^	750.30 ± 7.55^ab^	452.70 ± 9.77^b^	442.30 ± 4.84^b^
	WBCs (10^3^/ul)	8.71 ± 0.14	8.64 ± 0.11	15.13 ± 0.12^a^	13.41 ± 0.19^ab^	8.61 ± 0.08^b^	8.52 ± 0.09^b^
Differential Count	Lymp. (10^3^/ul)	6.79 ± 0.13	6.84 ± 0.14	11.71 ± 0.16^a^	10.68 ± 0.10^ab^	6.86 ± 0.03^b^	6.87 ± 0.13^b^
	Mono. (10^3^/ul)	1.00 ± 0.06	0.97 ± 0.06	1.72 ± 0.07^a^	1.32 ± 0.08^ab^	0.95 ± 0.03^b^	0.92 ± 0.03^b^
	Gran. (10^3^/ul)	0.94 ± 0.03	0.91 ± 0.03	1.91 ± 0.02^a^	1.58 ± 0.02^ab^	0.90 ± 0.02^b^	0.92 ± 0.02^b^

**Table 2:** Effect of *M. oleifera* Leaves Extract after Incorporating Silver Nanoparticles (Ag-NPs) against Azoxymethane (AOM) Induced Colon Cancer on Different Biochemical Measurements in Rats

		C	*M. oleifera * nano-extract	Induced cancer	M. oleifera nano-extract		
					Pre-treated	Simult-treated	Post-treated
Liver Enzymes	ALT (U/L)	44.70 ± 0.83	45.20 ± 0.88	84.70 ± 0.83^a^	45.10 ± 0.66^b^	44.60 ± 0.60^b^	45.20 ± 0.85^b^
	AST (U/L)	173.57 ± 1.47	174.20 ± 1.62	268.00 ± 3.12^a^	170.40 ± 1.13^b^	169.40 ± 1.50^b^	168.90 ± 1.10^b^
	ALP (U/L)	84.00 ± 0.72	85.30 ± 0.63	131.30 ± 1.26^a^	84.40 ± 0.70^b^	83.90 ± 0.84^b^	83.40 ± 0.67^b^
Lipid Profile	TC (mg/dl)	75.70 ± 0.68	74.40 ± 0.73	96.10 ± 0.64^a^	74.20 ± 0.70^b^	74.90 ± 0.74^b^	74.50 ± 0.48^b^
	T.Gs (mg/dl)	64.61 ± 0.67	65.14 ± 0.54	93.52 ± 0.57^a^	64.60 ± 0.45^b^	64.96 ± 0.59^b^	64.41 ± 0.75^b^
Kidney Functions	Urea (mg/dl)	57.56 ± 1.19	58.05 ± 0.84	102.67 ± 1.75^a^	58.34 ± 1.00^b^	57.82 ± 0.69^b^	58.38 ± 1.09^b^
	Creatinin (mg/dl)	0.81 ± 0.01	0.81 ± 0.02	2.57 ± 0.04^a^	0.81 ± 0.01^b^	0.81 ± 0.01^b^	0.81 ± 0.02^b^
	Total Protein (g/dl)	8.48 ± 0.07	8.37 ± 0.06	4.91 ± 0.07^a^	8.38 ± 0.08^b^	8.41 ± 0.05^b^	8.35 ± 0.09^b^
	Albumin (g/dl)	3.43 ± 0.07	3.53 ± 0.06	1.27 ± 0.05^a^	3.45 ± 0.08^b^	3.49 ± 0.07^b^	3.48 ± 0.06^b^

**Table 3:** Effect of *M. oleifera* Leaves Extract after Incorporating Silver Nanoparticles (Ag-NPs) against Azoxymethane (AOM) Induced Colon Cancer on the Major Cellular Contents and Oxidative Stress Markers in Colon Tissues of Rats

		C	*M. oleifera *	Induced cancer	*M. oleifera *nano-extract		
			nano-extract		Pre-treated	Simult-treated	Post-treated
Major Cellular Contents	Glucose (nmol/ g wet tissue)	3.70 ± 0.04	3.62 ± 0.09	1.13 ± 0.02a	1.56 ± 0.03^ab^	3.57 ± 0.08^b^	3.73 ± 0.05^b^
	LDH (U/ g wet tissue)	1742.41 ± 6.08	1737.60 ± 5.12	1047.80 ± 11.24^a^	1049.20 ± 8.05^a^	1735.20 ± 4.25^b^	1738.60 ± 4.48^b^
	Ca (mmol/ g wet tissue)	0.17 ± 0.01	0.17 ± 0.01	0.96 ± 0.01^a^	0.82 ± 0.02^ab^	0.15 ± 0.01^b^	0.16 ± 0.01^b^
Markers of the Oxidative Stress	TAC (mM/L)	1.31 ± 0.04	1.34 ± 0.04	0.48 ± 0.01^a^	0.49 ± 0.02^a^	1.37 ± 0.04^b^	1.35 ± 0.02^b^
	CAT (U/gm)	45.25 ± 0.24	45.34 ± 0.78	10.37 ± 0.14^a^	11.08 ± 0.31^a^	43.67 ± 0.55^b^	44.35 ± 0.78^b^
	GPx (U/gm)	21.37 ± 0.14	22.28 ± 0.61	11.40 ± 0.17^a^	11.70 ± 0.20^a^	20.96 ± 0.53^b^	21.39 ± 0.52^b^
	LPO (nmol/ g wet tissue)	44.00 ± 0.89	44.60 ± 0.51	144.40 ± 1.40^a^	151.20 ± 5.42^a^	43.60 ± 1.08^b^	43.20 ± 0.97^b^
	TPC (nmol/ mg Protein)	10.43 ± 0.06	10.34 ± 0.11	24.14 ± 0.36^a^	23.67 ± 0.33^a^	10.39 ± 0.10^b^	10.40 ± 0.12^b^

**Figure 4:**
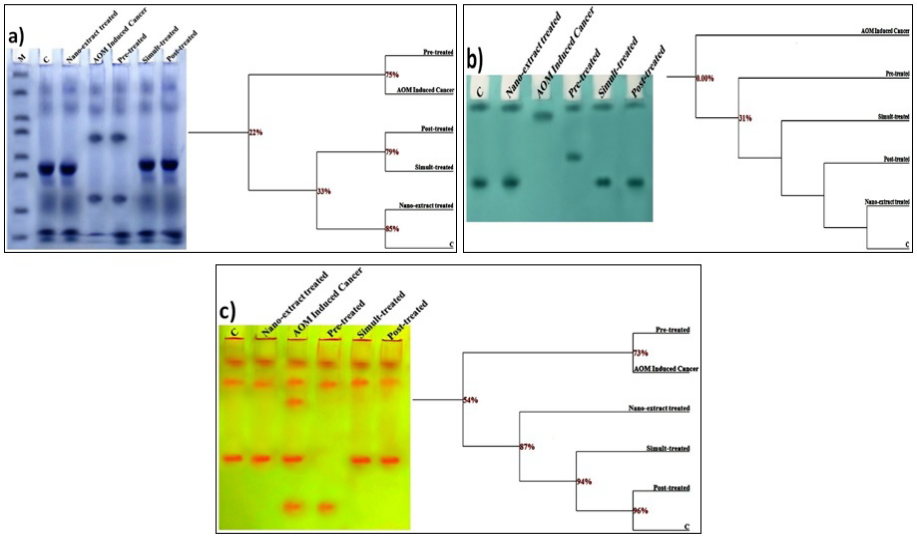
Native Electrophoretic Patterns Showing Physiological Effect of *M. oleifera* Leaves Extract after Incorporating Silver Nanoparticles (Ag-NPs) against Azoxymethane (AOM) Induced Colon Cancer on Bands Number, Arrangement and Similarity Percents on a) protein, b) lipid moiety of native protein and c) calcium moiety of native protein in colonic tissues of rats

**Table 4:** Effect of *M. oleifera* Leaves Extract after Incorporating Silver Nanoparticles (Ag-NPs) against Azoxymethane (AOM) Induced Colon Cancer on Relative Quantities of the Total Bands of Electrophoretically Separated Protein and Isoenzyme Patterns in Colonic Tissues of Rats

		C	M. oleifera nano-extract	Induced cancer	M. oleifera nano-extract		
					Pre-treated	Simult-treated	Post-treated
Native Protein Patterns	Native protein	82.95 ± 1.11	82.77 ± 1.13	59.62 ± 0.98^a^	68.71 ± 0.78^ab^	83.09 ± 1.54^b^	83.15 ± 0.95^b^
	Lipid moiety of native protein	41.23 ± 0.77	40.99 ± 1.11	18.87 ± 0.15^a^	41.12 ± 0.69^b^	40.96 ± 0.69^b^	41.25 ± 0.69^b^
	Calcium moiety of native protein	28.52 ± 0.32	28.71 ± 0.65	58.94 ± 0.63^a^	28.90 ± 0.51^b^	29.20 ± 0.78^b^	28.67 ± 0.42^b^
Native Isoenzyme Patterns	CAT	38.10 ± 0.81	38.03 ± 1.04	17.88 ± 0.16^a^	38.29 ± 0.48^b^	38.31 ± 0.91^b^	38.50 ± 0.89^b^
	POX	30.43 ± 0.30	30.52 ± 0.42	17.61 ± 0.42^a^	30.35 ± 0.29^b^	30.52 ± 0.83^b^	30.65 ± 0.41^b^
	α-EST	30.39 ± 0.42	30.60 ± 0.68	30.72 ± 0.49	30.48 ± 0.42	30.61 ± 0.40	30.49 ± 0.54
	β-EST	65.85 ± 0.29	65.32 ± 0.30	44.96 ± 0.15^a^	65.58 ± 0.36^b^	65.83 ± 0.59^b^	65.50 ± 0.37^b^

**Figure 5:**
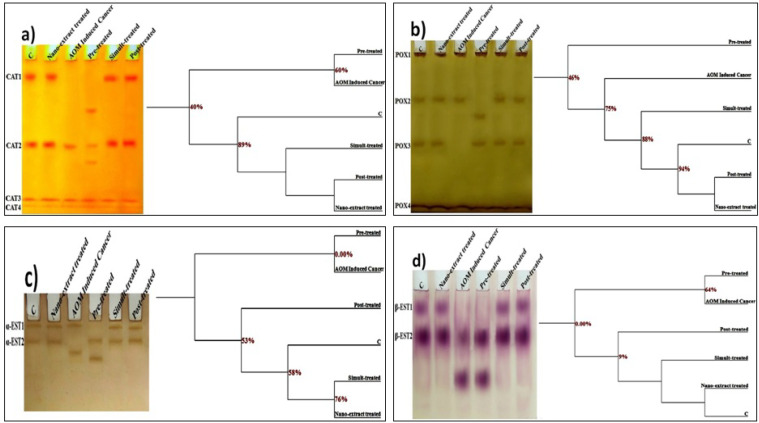
Native Electrophoretic Isoenzyme Patterns Showing Physiological Effect of *M. oleifera* Leaves Extract after Incorporating Silver Nanoparticles (Ag-NPs) against Azoxymethane (AOM) Induced Colon Cancer on Bands Number, Arrangement and Similarity Percents on a) Catalase (CAT), b) Peroxidase (POX), c) α-esterase (α-EST) and d) β-esterase (β-EST) in colonic tissues of rats

**Figure 6:**
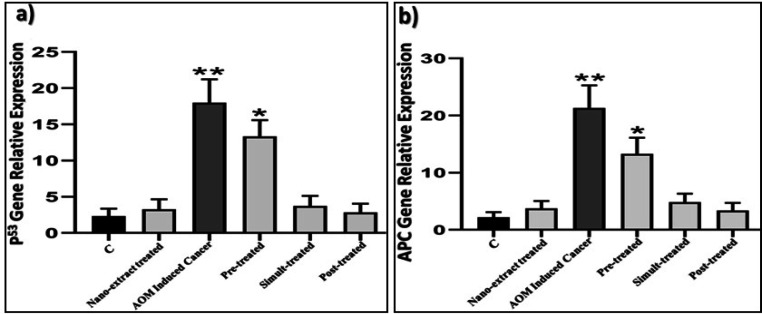
The Ameliorative Effect of *M. oleifera *Leaves Extract after Incorporating Silver Nanoparticles (Ag-NPs) against the Deleterious Effect of Azoxymethane (AOM) Induced Colon Cancer on a) level of Tumor Protein P53 (TP53) gene expression and b) level of Adenomatous Polyposis Coli (APC) gene expression in rats.

## Discussion

Carcinogenesis is conventionally defined by three stages: initiation, promotion and progression. The normal cells experience DNA damage (initiation stage). The initiated cells affect normal cells forming preneoplastic lesions (promotion stage). The malignant tumors develop finally leading to metastasis (progression stage) (Martín et al., 2016; Willis et al., 2017). It is well known that the synthetic chemotherapeutic agents have the ability to modify the intracellular pathways to slow, block or reverse the carcinogenesis (Johnson and Mukhtar, 2007). The present study was designed to develop novel strategies for treatment of colon cancer by mean of green nanotechnology through incorporating Ag-NPs into plant extract to enhance its biological efficiency as suggested recently by Aboulthana et al., (2019).

The haematological parameters were assayed due to the effective role of immune system in controlling tumor development to reveal relation of blood constituents to colon cancer and for explaining the blood relating functions of plant extract (Ashafa et al., 2009). The haematological alterations occurred mainly as a result of AOM injection attributed to the oxidative stress and attack of the free radicals that closely related to cancer progression (Childress, 2012). During the present study, it was noticed that RDW level elevated significantly in colon cancer induced group. This agreed with Yang et al., (2018) who postulated that the RDW level is closely related to prolonged inflammatory processes in the active malignancy and metastasis of colorectal cancer. The MPV is an early marker of platelet activation that consequently plays a key role in inflammation and atherothrombosis. Its level elevated significantly in colon cancer induced group and this was in agreement with Li et al., (2014) who postulated that elevation on MPV level attributed to its positive correlation to stage of tumor-nodule-metastases. Also, the WBCs and their differential count (Lymp., Mono. and Gran.) increased in colon cancer induced group due to stimulating the bone marrow through the inflammatory reactions to produce more leukocytes to migrate to inflammation site and this process was facilitated by PLT (Vieira-de-Abreu et al., 2012). *M. oleifera* nano-extract restored levels of hematological measurements to normal values in the simult- and post-treated groups and this was in agreement with Dhar and Gupta (1982) who reported that leaves of this plant are rich in various vitamins and Fe in significant amount sufficient to improve the blood status of the body.

Activities of the ALP, AST and ALP enzymes elevated significantly in colon cancer induced group and this agreed with Megaraj et al., (2014) who postulated that AOM induced hepatotoxicity and liver injury. This might refer to effect of the alkyl free radicals and the carbonium ions that belong to the highly reactive electrophiles targeting liver tissue and consequently leads to loss of membrane integrity and hence increasing membrane permeability. Therefore, these cytoplasmic enzymes leak at early stages of liver damage from hepatocytes into blood stream (Moharib et al., 2014). The nano-extract restored activities of the liver enzymes to normal levels and this might be attributed to presence of wide range of the polyphenolic compounds that increased after incorporating Ag-NPs as supported by Shousha et al. (2019) who emphasized that *M. oleifera* nano-extract contains higher concentrations of the active ingredients that exhibit antioxidant activity when compared to the crude one. Consequently, improvement towards normalization of the biomarker enzymes following nano-extract administration suggested that the extract exhibited functions in preserving structural integrity of hepatocellular membrane against ROS generated as a result of AOM injection.

Urea and creatinin were considered as indicators of kidney function and index of kidney dysfunction (Garba et al. 2007). In the present study, it was noticed that AOM caused renal toxicity represented by raising their concentrations and this might refer to effect of AOM that belongs to the hydrazine compounds that up-regulate biosynthesis of amino acids by stimulating urea cycle for removing excess ammonia derived from elevated amino acids (Liao et al., 2012). Moreover, urea level might be elevated as a result of converting amino groups (N-terminus) of amino acids into urea through urea cycle (Bando et al., 2011). Also, levels of the renal measurements might be increased with lowering TP and albumin levels due to the severe inflammatory and necrotic effects induced in kidney tissues by AOM injection (Tan et al., 2015). Levels of TP and albumin might be declined as a result of elevating levels of urea and creatinin that act as an inhibitor of protein synthesis (Yazar and Baydan, 2008) and / or the liver injury induced by AOM and consequently leads to the alterations in protein biosynthesis (Gürocak et al., 2013). *M. oleifera* nano-extract restored levels of the renal measurements to normal levels in all nano-extract treated groups. This was in accordance with Ameh and Alafi (2018) who reported that *M. oleifera* extract ameliorated the decline in protein content probably by scavenging ROS and improving the antioxidative status. Also, it might be attributed to presence of high concentrations of phenolic compounds that play an effective role in improvement of renal physiology and possess diuretic and natriuretic properties, as well as exerting the renoprotective effects that the antiapoptotic and antifibrotic properties of theses active constituents (Vargas et al., 2018). In addition, the TP and albumin levels restored to normalcy due to the beneficial effect of *M. oleifera* nano-extract on liver tissues that became able to biosynthesize protein normally.

Lipids are important components in the cell membranes for maintaining their architecture (Chung and Jung, 2003). T.Gs are the principal storage form of energy (Liu and Yang, 2003). In the present study, it was found that AOM caused significant elevation in levels of TC and T.Gs. This agreed with Yang et al., (2013) who showed that colorectal polyps were significantly associated with increased the TC and T.Gs levels. This might be attributed to various biochemical mechanisms proposed for the link between serum lipids and colorectal polyps. Moreover, the hypercholesterolemia is associated with the hyperinsulinemia and insulin resistance that can induce risk of the colorectal cancer (Borena et al., 2011). The carcinogenic substances that induce cancer cause defective lipids degradation or abnormal lipid synthesis. Also, the lipid measurements might be elevated due to deficiency of the lipoprotein lipase that stimulates the T.Gs uptake (Yuvaraj et al., 2007). Also, hypertriglyceridemia occurs due to the tumors growth and it is strongly associated with metastatic conditions (Nalini et al., 2006). The nano-extract lowered levels of these lipid measurements due to efficiency of the active constituents in nano-extract to develop lipolysis-stimulating agent in adipocytes (Holm, 2003).

CEA and CA19-9 exhibit their functions as a regulator for innate immune system, promoter for cellular aggregation and mediator for signal transduction. Therefore, they play an important role in tumor invasion and metastasis (Li et al., 2010). Their levels are considered as the most common biomarker for CRC and characterized tumor-associated antigens in both biochemical and clinical aspects (Ogata et al., 2009). Moreover, their levels are used for preoperative assessment of extent and outcome of cancer in addition to the postoperative monitoring of recurrence (Nakatani et al., 2012). In the present study, it was found levels of CEA and CA19-9 were elevated significantly in AOM induced colon cancer group. This agreed with Mare et al. (2013) who reported that AOM was categorized as a potent carcinogen that stimulates the oxidative stress and produces excess of ROS that cause metabolic instability in colon tissue leading to different changes in these tumor markers. Also, their levels were significantly correlated with lesion size and multiplicity of adenomas (Kim et al., 2017). The nano-extract decreased levels of these tumor markers and this might attributed to presence of the active ingredients and several secondary metabolites that are characterized by their antioxidant and anti-tumor activities (Kumari and Jain, 2012). Their anti-tumor and antioxidant efficiency (free radicals scavenging activity, reducing power and total antioxidant capacity) increased after incorporating Ag-NPs (Shousha et al., 2019).

The CRP is a positive protein in the acute phase of tissue damage and inflammation and elevation of its level reflected the ongoing inflammation and tissue damage much more accurately than other biochemical parameters (Hall et al., 2013). In the present study, AOM caused significant elevation in CRP level and this was in accordance with Rajendiran et al., (2018) who documented that CRP level in serum is significantly correlated to severity of colon inflammation and also this might be attributed to the inflammatory effect of the DSS that ingested via drinking water in combination with AOM during the induction process. Furthermore, the MPO activity is used as a good index of tissue injury induced by the inflammatory reactions (DOdorico et al., 2001). During the current study, the elevation in MPO activity colon cancer induced group attributed to the inflammatory effect of AOM that was generally associated with oxidative stress (Lowes et al., 2013) and / or due to the neutrophil infiltration that is linearly related to the MPO activity that increased as a result of effect of DSS ingested in combination with AOM (Pandurangan et al., 2015). *M. oleifera* nano-extract decreased levels of the inflammatory markers and this in accordance with Tad and Dass (2019) who emphasized that the anti-inflammatory effect of the plant extract against intestinal inflammation might be attributed to presence of the secondary metabolites such as alkaloids, flavonoids, terpenoids and saponins that well known by their ability to inhibit the inflammatory reactions. Incorporation of Ag-NPs into plant ex¬tract showed higher antioxidant capacity. Correlation between total phenolic con¬tent and antioxidants capability showed the strong influence of phenolic compounds in the antioxidant activity of Ag-NPs and leaves extracts as well.

LDH is one of the important enzymes catalyzing glycolytic pathway. Under normal conditions, it produced in the body in little amounts and increases in the tissues as a result of physiological abnormalities. Therefore, it is used as an indicator for incidence of cancers (Erez et al., 2014). During the current study, glucose level decreased significantly with lowering LDH activity in colon of cancer induced group. This agreed with Denko (2008) who suggested that LDH activity decreased due to increasing number of the cells that produce energy through glycolysis under anaerobic conditions by consuming great glucose amount during cancer development and therefore, the LDH consumed and hence its activity decreased. Also, Pujara and Chaudhary (2017) suggested that LDH activity decreased in the cancer tissue due to destruction of the growing cancer cells to other tissues leading to release of intracellular enzymes into blood stream and hence its activity decreased.

Calcium belongs to the trace elements that change in colon cancer and their levels have prognostic significance in its incidence and progression (Emel et al., 2005). In the present study, calcium level increased in colon tissues of cancer induced group. This might be attributed to the oxidative stress and the disturbances of calcium homeostasis that might be related to injury of the colon tissues during the induction process (Wulf, 2000). The nano-extract restored all altered measurements (glucose level, LDH activity and calcium level) assayed in colon tissues to normal values. This might refer to efficiency of the active constituents to inhibit the oxidative stress and the metabolic intermediates and hence inhibit generation of the ROS during activation of the microsomal enzyme (Waly et al., 2014).

Malondialdehyde (MDA) is the most common end product of polyunsaturated fatty acids peroxidation that induced by oxidative stress (Al-Henhena et al., 2015). In the present study, the MDA level elevated significantly in colon of cancer induced group. This was attributed to ability of AOM to initiate oxidative stress through producing extremely ROS after its metabolism in the body (Lahouar et al., 2014). Furthermore, the reactive metabolites increased during the hepatic AOM metabolism and/or during the process of colon carcinogenesis well. Consequently, these metabolites stimulate production of substantial amount of H2O2 in colon tumor cells then released into the circulation (Khan and Sultana, 2011). Also, the MDA induced protein damage, DNA fragmentation, gene mutations and loss of membrane integrity (Anilakumar et al., 2010). Therefore, the lipid and protein oxidation products increased significantly in AOM injected group during induction of the colon cancer.

During the current study, it was found that AOM caused significant decrease in TAC and activities of the antioxidant enzymes (CAT and GPx). This agreed with Burlamaqui et al., (2013) and supported by Waly et al., (2016) who suggested that AOM is able to modify activities of the antioxidant enzymes that provide the first line of cellular defense for eliminating the free radicals and play a critical role in maintaining the body’s defense mechanism against the destructive effects of ROS. *M. oleifera* possesses antioxidant activities due to presence of many biologically active phyto-constituents (Cabardo and Portugaliza, 2017). Treatment with Ag-NPs green synthesized with *M. oleifera* leaves extract could protect cells from damage, demise and dysfunction that caused by the oxidative stress. This might be attributed that nano-extract is capable to protect cells by stabilizing the membrane permeability through inhibiting peroxidation reactions and preventing glutathione depletion. The antioxidant profile was restored by the nano-extract which prevented the peroxidation reactions and generation of ROS in colinic cells as presented by its ability to quench and scavenge production of the free radicals as supported by Shousha et al., (2019). In addition, our findings were supported by Dhanapal and Ping (2017) who revealed that the antioxidant capacity of *M. oleifera* leaves extract increased after incorporating Ag-NPs. The improved antioxidant ac¬tivity might be related to the ability of antioxidant functional groups to adsorb onto surface of Ag-NPs.

Animal models were used for studying pathogenesis of colorectal tumors, as well as for evaluating efficiency of the extracts to be used as alternative treatments (De-Souza and Costa-Casagrande, 2018). In the current experiment, the colonic tissues were stained by H&E to be examined histopathologically. Throughout the colonic mucosa of the AOM-treated rats, severe histological abnormalities were noticed. This was in accordance with Sengottuvelan et al., (2006) and supported by Saleem et al., (2015) who reported that the oxidative stress that induced as a result of ROS generation with depletion of the antioxidant enzymes were responsible for inflammatory cells infiltration and the severe histopathological alterations noticed in colon cancer induced group. The alterations in architecture of colon mucosa with typical inflammatory changes in colonic architecture such as crypt and surface epithelial loss as well as infiltration of inflammatory cells and complete destruction of the epithelial architecture might be attributed to effect of DSS that administrated to rats through drinking water during the induction process (Huang et al., 2010). Also, these histopathological abnormalities were related to the antioxidant status of the colonic tissue that was altered as a result of excessive ROS production (Camacho-Barquero et al., 2007). Nano-extract minimized severity of the inflammatory responses that is considered as stage of colon cancer and malignancy criteria in nano-extract simult-treated group and prevented it completely in the post-treated group as compared to the other nano-extract treated ones. This might be attributed to presence of the phenolic compounds that accumulate in response to a wide range of stressors to exhibit their beneficial effect through their capability of singlet oxygen-quenching and radical scavenging activity (Mise et al., 2011). The nano-extract protected the colonic cells from damage and dysfunction that induced by oxidative stress due to incorporation of Ag-NPs that increased the biological efficiency and the antioxidant activity of plant extract (Mitiku and Yilma, 2017 ; Shousha et al., 2019).

Electrophoresis used as standard tool for separating, identifying and quantifying of different proteins based on net charge, size and shape of the protein molecules. Therefore, it is used for analyzing stoichiometry of a specific subunit of a protein complex (Shah et al., 2010). Each band is made up of group of the proteins that characterized by independent metabolic properties (O’Connell et al., 2005). The electrophoretic alterations may occur at qualitative level by hiding one or more of normal bands with existence of abnormal ones. Otherwise, the alterations may occur at quantitative level by altering quantities of the bands that identified at normal mobilities. The SI is only related to the physiological state and indicates the qualitative alterations and it is inversely proportional to the GD. The low SI values are associated with high GD values indicating differences in number and arrangement of the bands (Abdalla et al., 2015 ; Sharada et al., 2015; Aboulthana et al., 2016). During the current study, it was found that AOM caused alterations in native protein pattern. This was in agreed with Yasui and Tanaka (2009) who suggested that the native protein might be altered due to its deleterious effect on RNA transcripts, altering their amounts, localization or translation and / or due to its direct interaction of AOM with proteins. Moreover, the modifications in protein pattern might be related to altering DNA organization and hence changing DNA function and hence protein expression (Aboulthana et al., 2020 ; Deabes et al., 2021a). Lipids are considered as one of the most sensitive cellular macromolecules (Javed et al., 2014). In the present study, the native lipoprotein pattern was altered severely during induction of colon cancer as a result of AOM injection. This might be attributed to accumulation the peroxidation products that attack the lipid portion through inducing formation of the ROS associated with depletion of the antioxidant defenses leading consequently to oxidative modifications and denaturation of the lipid moieties of proteins as suggested by El-Sayed et al., (2018) and supported recently by Aboulthana et al., (2020). Calcium-moiety of native protein categorized as low molecular weight acidic protein and it inhibits formation of hydroxyapatite leading to alterations in the mineralization process. In addition, it exhibits an effective role in maintaining integrity of the tissues against the toxic agents (Surowiak et al., 2007). During the present study, it was found that AOM caused alterations in calcium moieties of the native protein and this was in accordance with El-Sayed et al., (2018) who reported that the abnormalities occur in this native pattern qualitatively and quantitatively due to the oxidative stress and overproduction of the ROS that convert hydrogen atom (active) from these biologically active macromolecules. Moreover, these proteins might be altered due to the abnormal mineralization in the tissue (Abulyazid et al., 2017). As obtained during the current experiment and supported recently by Aboulthana et al., (2020), the alterations in this protein pattern might be related to elevating calcium concentration in the colon tissue and this leads to stimulating the oxidative stress and hence leads to variations in this protein pattern. *M. oleifera* nano-extract showed beneficial effect against the electrophoretic alterations in the different native protein patterns and this might refer to presence of the active polyphenolic constituents that increased after incorporating Ag-NPs (Shousha et al., 2019). 

Antioxidant enzymes are tissue dependent and vary from tissue to tissue. Their activities might be altered due to degeneration of the protein contents a result of attack of the ROS that consequently lead to changing the metabolic pathways (Ramanathan et al., 1999). In the present study, severe alterations were noticed in the electrophoretic antioxidant isoenzymes in colon of cancer induced group. This was in accordance with Aboulthana et al., (2020) who reported that ROS were responsible for changing rate of protein expression secondary to DNA damage leading to structural changes in protein portion of native enzymes. These isoenzymes remain electrophoretically integrated if there were no abnormalities in rate of the protein expression (Djordjevic et al., 2010 ; Abulyazid et al., 2017). Furthermore, the electrophoretic changes might occur due to binding of AOM to the native macromolecules inducing secondary structural changes in addition to effect of products of the oxidative stress and the peroxidation reactions (Mansouri et al., 2013). The *M. oleifera* nano-extract exhibited antioxidant efficiency against the abnormalities in the electrophoretic in CAT and POX patterns. This might refer to enhancing the total polyphenolic compounds that exhibit scavenging activity against ROS attack (Kumazawa et al., 2002).

Esterase-like albumin activity is considered as marker for many chronic diseases and expressed by several molecular forms with hydrodynamic properties and identified by their molecular weights (Thangthaeng et al., 2011). It can be visualized by staining the substrate (α- and β-naphthyl acetate) in the presence of dye coupler (Fast Blue RR salt) (Ahmad et al., 2012). In the present study, alterations were noticed in the electrophoretic EST pattern and this agreed with Deabes et al., (2021b) and Aboulthana et al., (2020) who confirmed that these changes might refer to oxidative stress and generation of the ROS that targeting structure of albumin and hence lead to various mutations occurred at qualitative level by hiding one or more of normal bands with existence of abnormal ones. Scavenging of the free radicals as hydroxyl radical which is the major cause of oxidation reactions by extract may be attributed to their phenolic compounds which are the primal source of antioxidant ability (Ahmed, 2010). The nano-extract exhibited an important role in maintaining integrity of EST isoenzymes against the oxidative reactions due to presence of these polyphenols that stimulate the antioxidant enzymes to overcome attack of the reactive species targeting these macromolecules (Fraga et al., 2010).

The irregularities in cell cycle are usually responsible for cancer progression (Moghadamtousi et al., 2014). AOM is activated in liver by Noxidation, generating reactive compounds essential for chemical carcinogenesis (metilazoximetanol ion and methyl diazoxide) being brought to the colon into the bloodstream or via bile as a glucuronide conjugate. After activation, the methylated DNA is mainly at positions N7-guanine and O6-guanine (Seike et al., 2006). Both of the TP53 and APC are the most sensitive genes susceptible to be mutated during incidence of colorectal cancers (Lynch and de la Chapelle, 2003). During the present study, it was noticed that the relative expression of* TP53* gene elevated significantly in AOM induced colon cancer group due to accumulation of TP53 protein within the tumor cells. Also, elevation of the *TP53* gene expression might refer to stability of TP53 protein that increased as a result of the aberrant alterations in acetylation and/or phosphorylation status of TP53 and consequently leads to suppressing it’s binding to DNA consensus sequences. Moreover, the post-translational modification is responsible for suppression of TP53 activity in colon of cancer induced (Minamoto et al., 2001 ; Nambiar et al., 2002). APC belongs to the most important tumor suppressor genes that expressed as a result of precancerous lesions during colon carcinogenesis and promote cellular differentiation in intestinal lineages (Xu et al., 2017). Elevation of the relative expression of *APC* gene was noticed in colon cancer induced group and this was in accordance with Grivennikov et al., (2012) who supported that level of *APC* gene expression was noticed greater in colon cancer induced group and this might be attributed to overexpression of the APC that is required as a central gatekeeper protein for regulating multiple pathways in colon cancer progression. Also, the changes in expression of the APC protein that is related to the alterations in expression of the *APC* gene lead to promoting colon cancer due to the abnormalities in cell cycle (Ghatak et al., 2017). *M. oleifera* nano-extract decreased the relative expression of TP53 and APC genes in the simult- and post-treated groups to normal levels. Although it could not decrease their levels in pre-treated group, it decreased their levels significantly as compared to cancer induced group. This might be attributed to efficiency of *M. oleifera* leaves extract to scavenge oxygen and nitrogen-based reactants generated in mitochondria, stabilizes mitochondrial membrane in addition to its ability to enhance anti-apoptotic signaling (El-khadragy et al., 2018). Incorporation of Ag-NPs into plant extracts enhanced the antioxidant properties through increasing the total antioxidant capacity, total reducing power and free radical scavenging activity in comparison with the crude extracts. Moreover, the nano-extract exhibited high cytotoxicity against growth of human colon cancer cells compared to the crude ones. This might be attributed to increasing concentrations of the active phyto-constituents that have the ability to inhibit cell proliferation and induce programmed cell death for controlling growth of the malignant cells (Aboulthana and Sayed, 2018 ; Aboulthana et al., 2019 ; Shousha et al., 2019).

In conclusion, Silver *M. oleifera* leaves nano-extract ameliorated the colon cancer induced by AOM through lowering levels of the hematological and biochemical measurements and restoring their levels to normal values in all nano-extract treated groups especially in simult- and post-treated groups. It decreased levels of the tumor (CEA and CA 19-9) and inflammatory (CRP and MPO) markers to normal levels in both of nano-extract simult- and post-treated groups. Moreover, it ameliorated the antioxidant system by elevating activities of the antioxidant enzymes (CAT and GPx) with lowering products of the lipid and protein oxidation reactions (LPO and TPC) in all nano-extract treated groups and restored their levels to normalcy in nano-extract simult- and post-treated groups and hence it prevented the inflammatory reactions detected histopathologically in colon tissues and restored the tissue to its normal configuration in that groups. As regard to the electrophoretic patterns, the nano-extract exhibited improvement at qualitative level through restoring the absent normal bands with hiding the abnormal ones. At the quantitative level, it restored the relative quantities of the total bands to normal values in the nano-extract simult- and post-treated groups. Also, the molecular assay supported the findings obtained by biochemical and electrophoretic assays and emphasized that the nano-extract decreased expression of the tumor suppressor genes (*TP53* and *APC*) and restored their expression levels to normalcy in nano-extract simult- and post-treated groups.

## Author Contribution Statement

W.G.S. and W.M.A. proposed the research idea and designed the experimental model for this work. A.H.S. and M.H.S. were responsible for preparing the plant extract. W.M.A., E.A.E. and M.H.S. performed the experimental work and provided reagents/materials necessary for experiments. A.H.S. was responsible for preparing the silver plant nano-extract. All authors collected the theoretical details from the previous studies, interpreted, analyzed the data and draft the manuscript then they read and approved the final manuscript. W.M.A. correspond the manuscript. 
